# Identification of the potential biomarkers associated with circadian rhythms in heart failure

**DOI:** 10.7717/peerj.14734

**Published:** 2023-01-20

**Authors:** Qiang Sun, Jun Zhao, Li Liu, Xiaoliang Wang, Xinshun Gu

**Affiliations:** 1 Department of Cardiology, The Second Hospital of Hebei Medical University, Shijiazhuang, China; 2 Department of Cardiology, The First Hospital of Qinhuangdao, Qinhuangdao, China

**Keywords:** Heart failure, Circadian rhythms, Biomarker, Bioinformatics analysis

## Abstract

**Background:**

Heart failure (HF) is a syndrome with multiple clinical symptoms resulting from damage to the heart’s structure and/or function with various pathogenic factors, which has developed as one of the most severe threats to human health. Approximately 13% of genes and about 8% of proteins contained in the heart are rhythmic, which could lead to HF if disrupted. Herein, we aimed to identify the circadian rhythms-related hub genes as potential biomarkers contributing to the identification and treatment of HF.

**Methods:**

Expression data of ischemic and dilated cardiomyopathy samples with or without HF were collected from the GEO database. First, genes with differential expression in HF and healthy samples were identified, named as differentially expressed genes (DEGs), which were then intersected with circadian rhythms-related genes to identify circadian rhythms-related DEGs. A protein-protein interaction (PPI) network was established to screen hub genes. The performance of the hub genes to identify HF among healthy controls was assessed by referring to the receiver operating characteristic (ROC) curve. Additionally, quantitative real-time polymerase chain reaction (RT-PCR) was run to further validate the hub genes depending on clinical human peripheral blood samples.

**Results:**

A total of 10,163 DEGs were determined, composed of 4,615 up-regulated genes and 5,548 down-regulated genes in HF patients in comparison to healthy controls. By overlapping the circadian rhythms-related genes in the Circadian Gene DataBase (CGDB), 723 circadian rhythms-related DEGs were obtained, mainly enriched in regulating lipid metabolic process, circadian rhythm and AMPK signaling pathway. Eight hub genes were screened out through the PPI network. The ROC curve indicated the high accuracy of five hub genes with AUC > 0.7, which also showed high accuracy validated by the external validation dataset. Furthermore, according to the results of quantitative RT-PCR, the HF group showed significantly increased relative mRNA expression of CRY2 and BHLHE41 while the decreased ARNTL and NPAS2 in comparison to controls, indicating the four hub genes as potential biomarkers of HF.

**Conclusion:**

Our study validated that ARNTL, CRY2, BHLHE41 and NPAS2 could serve as potential biomarkers of circadian rhythm in HF. These results may provide a reference for employing novel markers or targets for the diagnosis and treatment of HF.

## Introduction

Heart failure (HF) emerges as a syndrome with complex clinical symptoms elicited by the damage to the heart’s structure and/or function due to various pathogenic factors (McDonagh et al., 2021), which indicates the terminal stage of various heart diseases accompanied with a permanent deterioration in prevalence, incidence, mortality, and re-hospitalization rates. The prevalence of HF was reported as 1–3% in the general population among developed countries, and increased to about 5–9% in the elderly (van Riet et al., 2016; Virani et al., 2020).

Chronic heart failure (CHF) leading to disabling symptoms and high hospitalization rates, which exerts a heavy burden on patients, followed by their families, and society’s healthcare resources. Previous research has suggested a possible association of the disrupted circadian rhythm with the accelerated HF development, while the underlying mechanisms remain to be elucidated. All living organisms on the earth exhibit the circadian rhythms with a 24-h cycle, which are manipulated by the biological clock that provides basic timing information to manipulate biochemical, physiological, and behavioral processes with the adaptation to the environment and its variations. It has been demonstrated that approximately 13% of genes and 8% of proteins show rhythm in the heart, and disruptions of the molecular clock could lead to significant cardiovascular disease (Storch et al., 2002), which may also perturb the delicate balance with severe repercussions on HF outcomes (Alibhai et al., 2014; Song et al., 2022). As a result, a comprehensive elucidation of the link between circadian clock-related genes and the heart will contribute to developing a novel diagnostic and therapeutic approach to HF.

The current study was carried out to identify the circadian rhythms-related hub genes and evaluate their diagnostic value in HF, providing reference for detecting and treating the disease depending on the genomic expression profiles. Differentially expressed genes (DEGs) in HF in comparison to healthy samples were screened out according to the analysis datasets GSE57338 from the Gene Expression Omnibus (GEO). Subsequently, circadian rhythm-related genes were overlapped in the Circadian Gene DataBase (CGDB) to obtain the circadian rhythm-related DEGs. To explore the potential molecular mechanisms underlying at the pathogenesis of HF, we enriched the functions and pathways. The protein-protein interaction (PPI) network was used to analyze the rhythm-related DEGs, and the MCODE in Cytoscape software to identify hub genes. Hub genes also exhibited a high diagnostic accuracy according to the receiver operating characteristic analysis (ROC) validated in the external validation dataset. Eventually, we determined the mRNA expression levels of hub genes by quantitative RT-PCR in clinical samples of human peripheral blood. The rhythms-related hub genes for circadian may provide reference for potential biomarkers and therapeutic targets for HF.

## Materials and Methods

### Data source

GSE57338 and GSE5406 profiles were retrieved from the GEO database. The GSE57338 dataset, covering 136 controls and 177 HF samples, was employed for training. The GSE5406 dataset was employed as an external validation set composed of 16 controls and 194 HF samples. A total of 1,273 circadian rhythm-related genes were collected from the CGDB database (http://cgdb.biocuckoo.org).

### Acquisition of genes (DEGs) with differential expression

Data from microarrays were generally analyzed using R software after normalization. The DEGs of controls with HF samples were determined on the Limma R package with —log2FC—>0.5 and adj.P.Val<0.05 as the threshold (Love, Huber & Anders, 2014). A heatmap cluster and a volcano plot were created employing the “pheatmap” and “ggplots” packages in R software.

### Functional annotation and pathway enrichment analysis

The Gene Ontology (GO) function and Kyoto Encyclopedia of Genes and Genomes (KEGG) (Kanehisa & Goto, 2000; Chen et al., 2017) were employed on the “clusterProfiler” package for pathway enrichment analyses, among which biological processes (BP), molecular functions (MF), and cellular components (CC) were selected referring to the GO functional categories. Statistical significance was defined as Adj. *P* < 0.05.

### PPI network and module analysis

The protein interaction of circadian rhythms-related DEGs was analyzed according to the STRING (Szklarczyk et al., 2015). The confidence score set at the highest (0.900) was taken to screen out genes with strong interactions. The visualized PPI network was performed by Cytoscape (Shannon et al., 2003). Subsequently, MCODE, an open-source software tool, was used in Cytoscape to identify the significantly enriched modules from the PPI network based on module scores ≥ 5.

### Evaluation and validation of the diagnostic performance of hub genes

The hub genes’ diagnostic value was assessed by plotting ROC curves (Robin et al., 2011) employing the pROC package. The area under the curve (AUC) indicated the accuracy and diagnostic performance of hub genes.

### Sample collection

Twenty-one HF patients and ten controls in The First Hospital of Qinhuangdao from October 2019 to November 2020 were enrolled. The protocol has obtained the approval from the Medical Ethics Committee of The First Hospital of Qinhuangdao (ethical approval no.201902A084). The enrolled subjects were asked to submit the informed consent, which complied with the Declaration of Helsinki. Only HF with the decreased ejection fraction (left ventricular ejection fraction 40%) and an elevated N-terminal pro-brain natriuretic peptide (NTproBNP) > 200 pg. per millilitre in patients was involved. Patients for newly diagnosed myocardial infarction within 12 months or advanced HF requiring ongoing treatment were excluded. In addition, the control group was matched with ten subjects without HF. The Trizol method (Rio et al., 2010) was employed to extracted the total RNA of human peripheral blood. The NanoDrop 2000 Spectrophotometer (Thermo Fisher Scientific, Waltham, MA, USA) was employed to quantify the concentration and purity of the extracted RNA. cDNA synthesis was achieved by employing Hifair III 1st Strand cDNA Synthesis SuperMix (Yeasen Biotech, Shanghai, China). Quantitative RT-PCR was run referring to SYBR Green Master Mix (Yeasen Biotech, Shanghai, China) and conducted in the CFX connect quantitative real-time PCR detection system (Bio-Rad, Hercules, California, USA). Primer sequences for ARNTL, NPAS2, BHLHE41, CRY2 and PER3 were purchased from Tianyi Huiyuan Biotech (Beijing, China), shown in Table S1. Beta-actin was employed as an internal gene, and the expression level of mRNA relative to control was calculated by using the 2^−ΔΔcycle threshold (CT)^ method.

### Statistical analysis

The measurement data were described as mean ± standard, median (interquartile range) or percentage (%). Student’s *t*-test was performed to compare nonpaired for normally distributed variables between the HF and controls group, and the Mann–Whitney U test for non-normally distributed variables, the chi-square test for categorical variables on SPSS 23.0 (IBM, Armonk, NY, USA). *P*-values below 0.05 represents significant differences.

## Results

### Identification of differentially expressed genes (DEGs)

**Figure 1:**
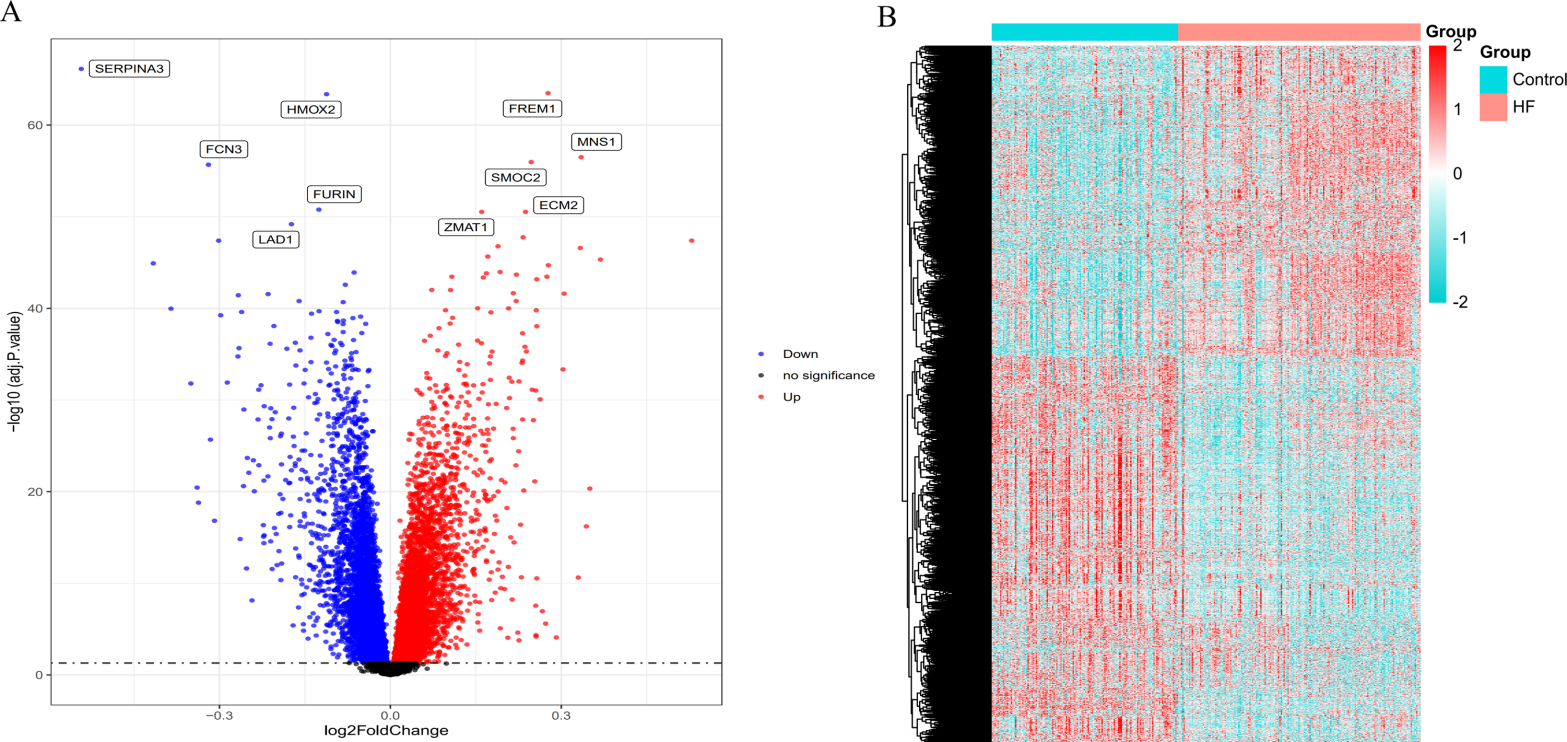
Volcano plot and heatmap depict the differences in gene expression between HF samples (*n* = 177) and controls (*n* = 136). (A) Volcano plot of genes identified in HF. Up-regulated DEGs (red, *n* = 4615); Down-regulated DEGs (blue, *n* = 5548); No difference (Black). (B) Heat maps of differentially expressed genes. The ordinate represents the sample, and the abscissa represents the differentially expressed gene (*n* = 10163). The color represents the expression level of the gene. The redder the color, the higher the gene expression level in samples of HF patients. The bluer it is, the lower the gene expression level in patients with HF.

According to the standardized microarray expression data, 10163 DEGs were identified in  GSE57338, covering 4,615 up-regulated and 5,548 down-regulated genes (Fig. 1A), with the expression profile displayed in the heatmap in HF samples (*n* = 177) compared to controls (*n* = 136) (Fig. 1B).

### GO and KEGG enrichment analysis of circadian rhythms-related DEGs

**Figure 2:**
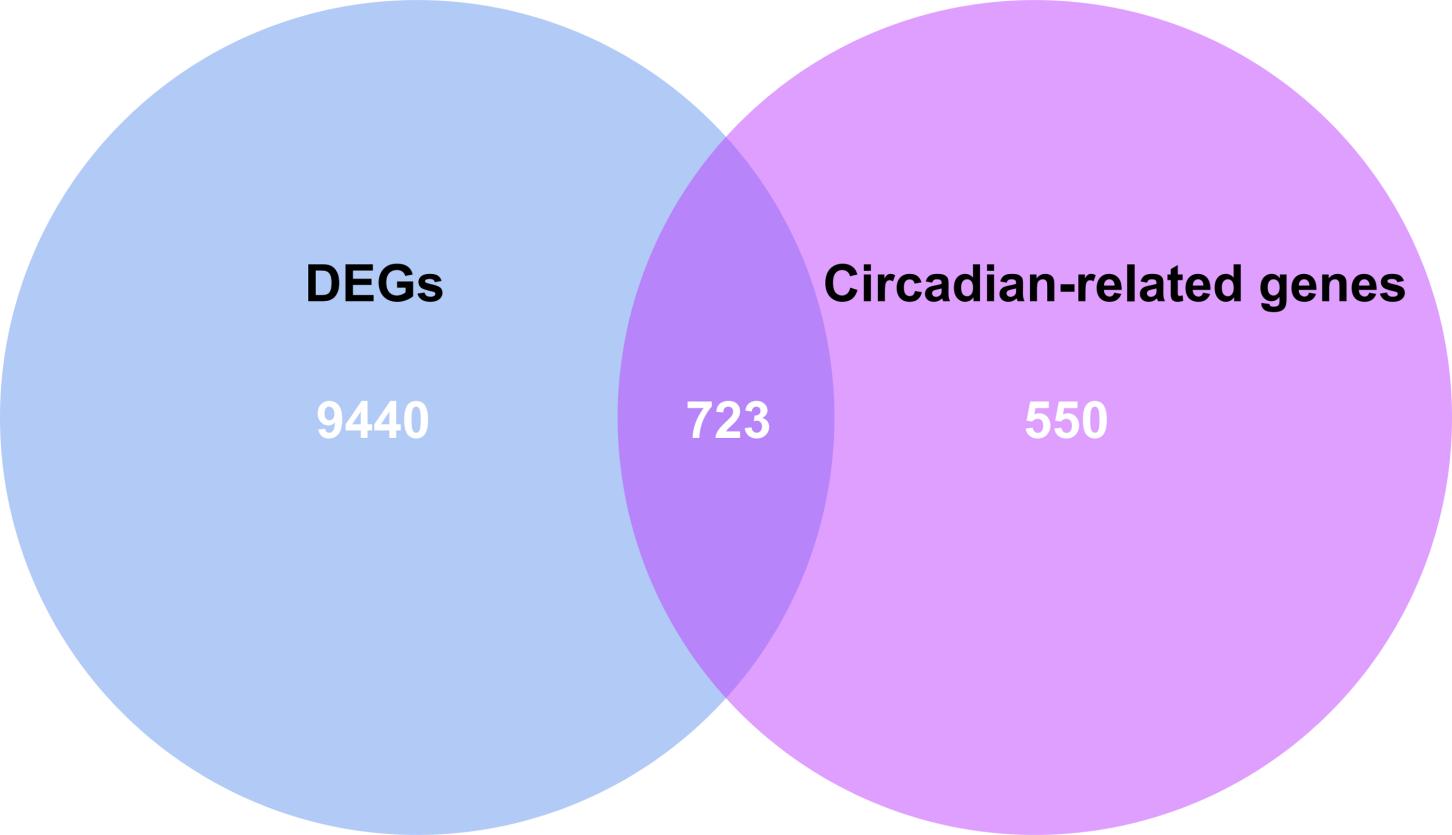
Venn diagram of the intersection of circadian rhythm-related genes and differentially expressed genes. The blue area represents the differentially expressed genes in HF (*n* = 177) compared with the control (*n* = 136), the purple area represents the circadian rhythm-related genes, and the overlapping part for the differentially expressed circadian rhythm-related genes.

**Figure 3:**
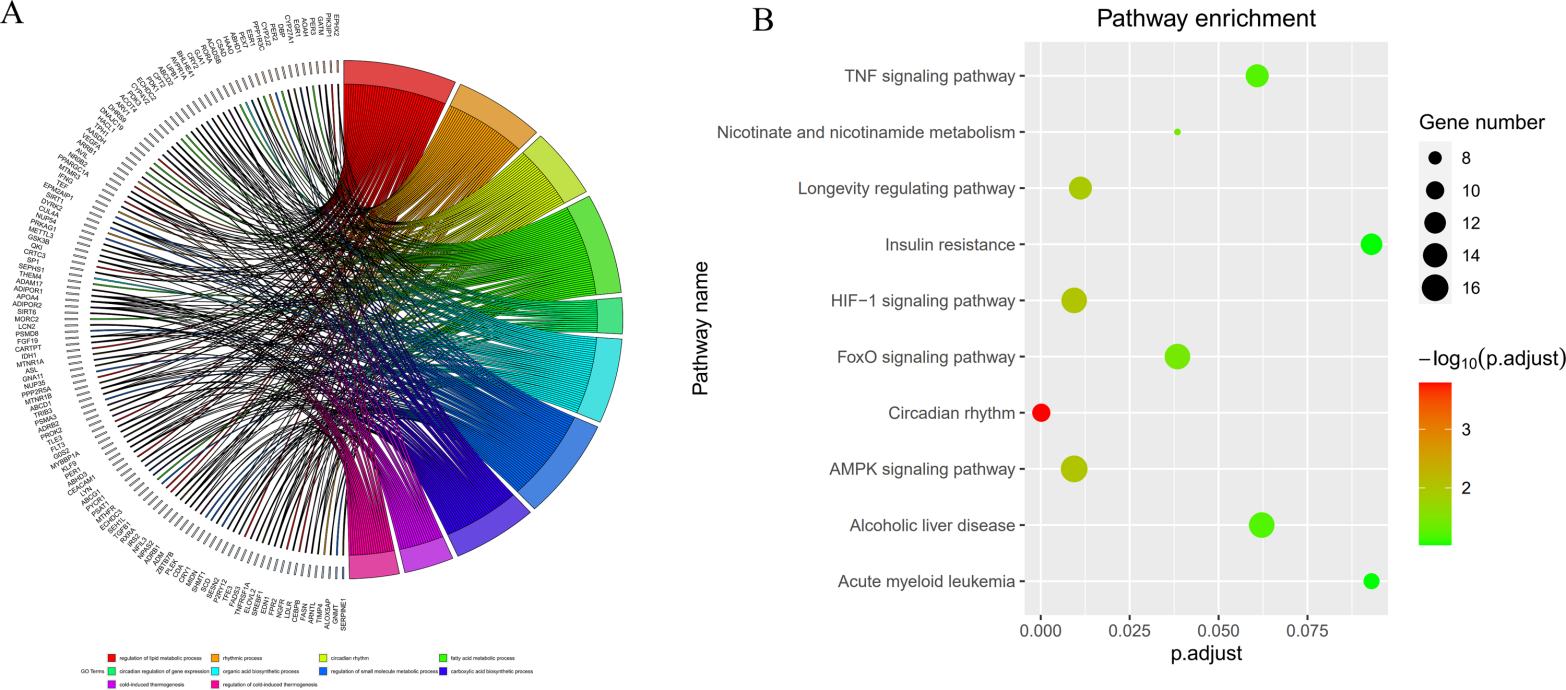
Circadian rhythm-related DEGs analyzed by GO and KEGG. (A) Circle diagram of GO showed circadian rhythms-related DEGs enriched in different biological functions. (B) KEGG pathway enrichment scatterplot.

By intersecting the circadian rhythms-related genes with DEGs, a total of 723 circadian rhythms-related DEGs were determined (Fig. 2), with the top 10 results of GO functions depicted in Fig. 3A. The GO category indicated the prominent enrichment of circadian rhythms-related DEGs in functions of manipulating lipid metabolic process, rhythmic process and circadian rhythm. KEGG analysis demonstrated the role of circadian rhythms-related DEGs in the Circadian rhythm, AMPK pathway and HIF-1 pathway (Fig. 3B).

### PPI network construction and module analysis

**Figure 4:**
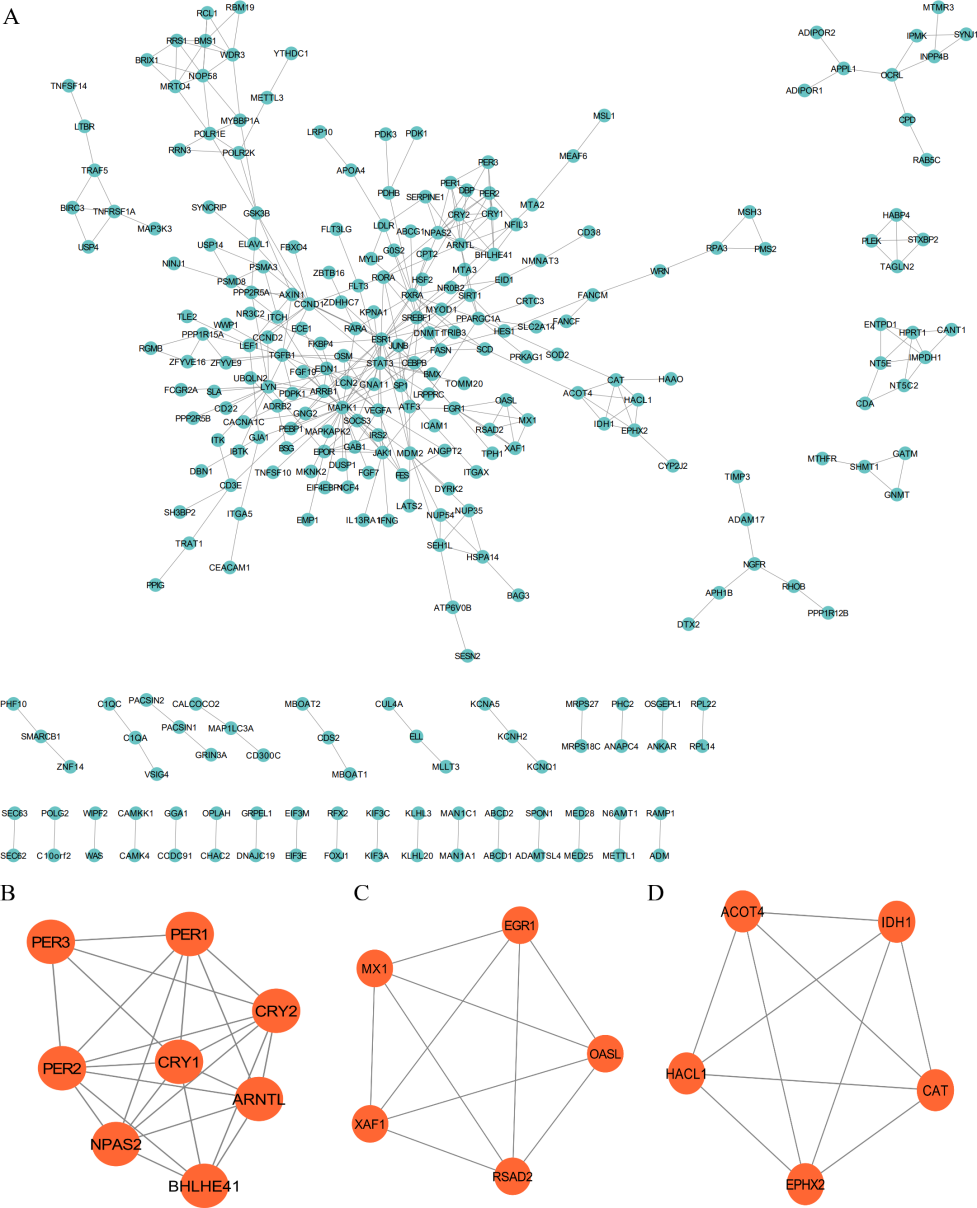
Protein-protein interaction (PPI) and MCODE analysis of differentially expressed proteins. (A) Proteins and interactions of PPI network. (B) MCODE1 (score = 6.857). (C) MCODE2 (score = 5). (D) MCODE3 (score = 5).

The PPI network was employed to examine the circadian rhythm-related DEGs, through which 278 nodes and 404 protein interactions were identified (Fig. 4A). Afterwards, MCODE was employed to extrude the prominent module in the PPI network, which isolated three core modules. In detail, module A (score = 6.857) covered 24 interactions and eight protein nodes (Fig. 4B), module B (score = 5) for ten interactions and five nodes (Fig. 4C), and module C (score = 5) for ten interactions and five nodes (Fig. 4D). The eight MCODE Score Top1 module genes, CRY1, ARNTL, PER2, BHLHE41, CRY2, PER3, NPAS2 and PER1 were selected as hub genes for further analysis.

### Identification of hub genes

**Figure 5:**
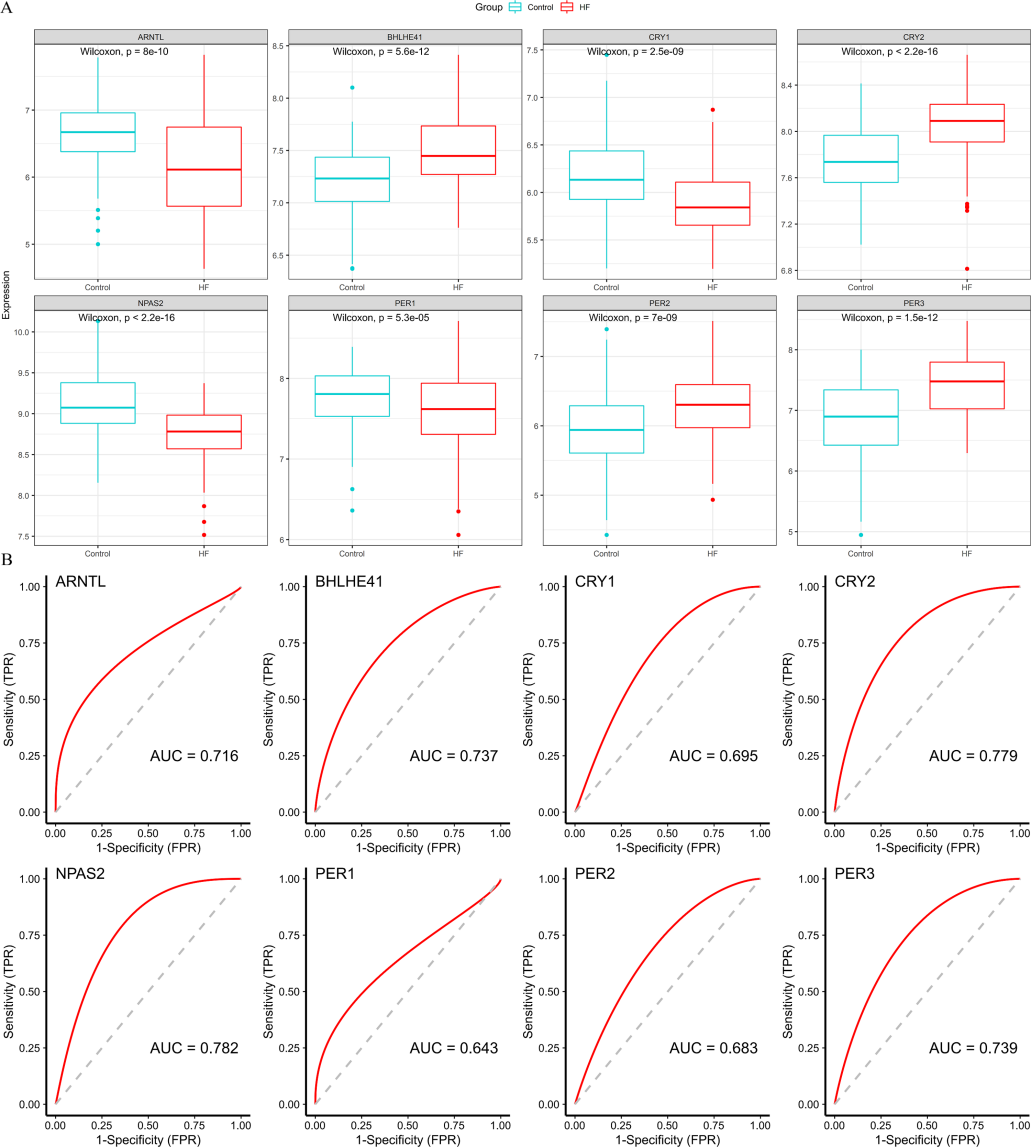
Validation of hub genes based on the training set. (A) The box plots showed the hub genes expression of each group in the training set. Red boxes (HF group, *n* = 177), blue boxes (control group, *n* = 136). (B) ROC curves for evaluating the diagnostic values of eight hub genes.

Four down-regulated hub genes (ARNTL, CRY1, NPAS2 and PER1) and four up-regulated hub genes (PER2, PER3, CRY2 and BHLHE41) were identified in HF samples (Fig. 5A). According to the ROC curves, the genes of ARNTL, PER3, CRY2, BHLHE41 and NPAS2 exhibited high accuracy with AUC > 0.7 (Fig. 5B).

**Figure 6:**
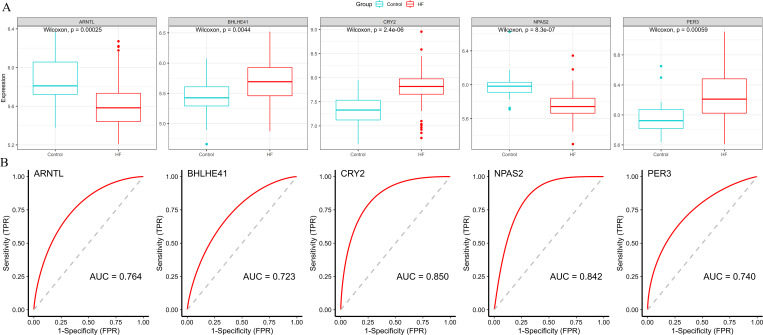
Validation of hub genes in the external validation set. (A) The box plots showed the hub genes expression of each sample in the external validation set. Red boxes (HF group, *n* = 194), blue boxes (control group, *n* = 16). (B) ROC curve for the hub genes in the external validation set.

Subsequently, we validated the expression and diagnostic value of ARNTL, PER3, CRY2, BHLHE41 and NPAS2 in GSE5406. The genes in GSE5406 exhibited a similar expression trend to those in the training set GSE57338 (Fig. 6A), with a more prominent AUC values over 0.7 (Fig. 6B). As a result, ARNTL, PER3, CRY2, BHLHE41 and NPAS2 were identified as potential diagnostic markers for HF.

### Baseline characteristics of participants

Twenty-one HF patients and ten without HF were enrolled, whose demographic, clinical characteristics, medication use and laboratory parameters are provided in Table 1. No significant differences were found in baseline characteristics between the HF group and the control group in terms of age, gender, heart rate, blood pressure, and medical history, as well as in medication use, except for the higher rate of ACE inhibitors or ARBs drug use in the HF group in comparison to the control (*P* = 0.002). The HF group exhibited significantly decreased LVEF (*P* < 0.001) and increased levels of NT-pro-BNP (*P* < 0.001).

**Table 1:** Characteristics of participants.

Variables	Heart failure (*n* = 21)	Control (*n* = 10)	*p*-value
Demographic characteristics	
Age, year	71.095 ± 11.269	68.500 ± 10.212	0.478
Male (%)	12 (57.1)	7 (70.0)	0.697
Vital signs on admission			
Heart rate, beats/min	68.000 (63.500–88.500)	68.000 (65.000–82.250)	0.932
Systolic BP, mm Hg	131.000 (122.500–155.000)	127.000 (121.000–147.500)	0.642
Diastolic BP, mmHg	68.000 (64.500–86.000)	71 (67.500–85.250)	0.525
Accessory examination			
LVEF (%)	32.429 ± 4.296	61.800 ± 3.490	<0.001
NT-pro-BNP (pg/mL)	4500.320 (3038.170–7693.585)	15.5000(0–57.890)	<0.001
Medical history			
Myocardial infarction (%)	4 (19.0)	1 (10.0)	1.000
Hypertension (%)	8 (38.1)	2 (20.0)	0.428
Atrial fibrillation (%)	3 (14.3)	2 (20.0)	1.000
Diabetes mellitus (%)	6 (28.6)	2 (20.0)	1.000
Medications			
Beta-blockers (%)	14 (66.7)	3 (30.0)	0.121
ACE inhibitors or ARBs (%)	17 (81.0)	2 (20.0)	0.002
Diuretics (%)	8 (38.1)	1 (10.0)	0.205
Statins (%)	10 (47.6)	4 (40.0)	1.000

**Notes.**

BP blood pressure LVEF left ventricular ejection fraction NT-pro-BNP N-terminal pro-B-type natriuretic peptide ACEI angiotensin-converting enzyme inhibitors ARB angiotensin II receptor blockers

### Validation of hub genes

**Figure 7:**
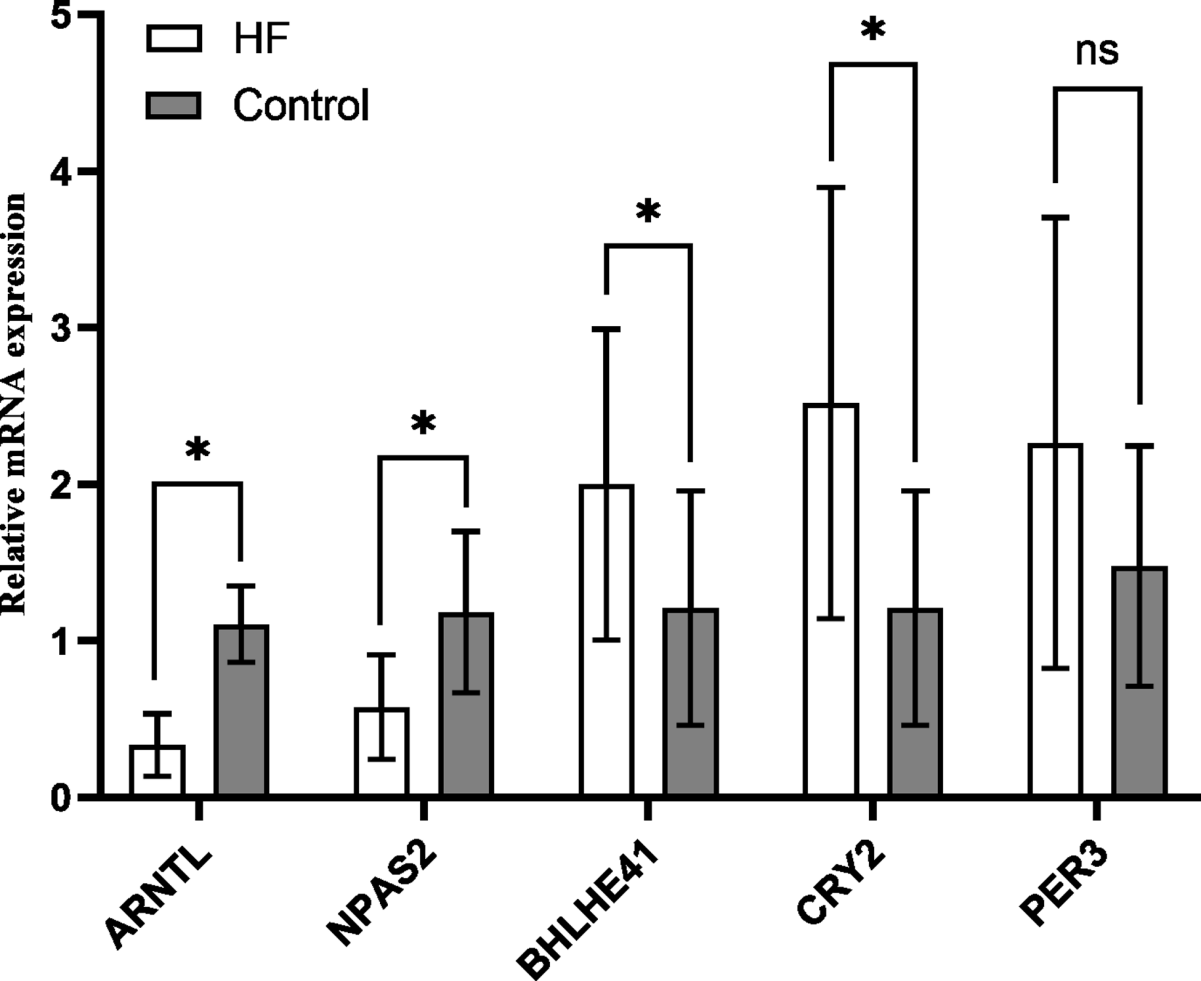
Validation of hub genes in the peripheral blood of the participants. Relative mRNA level of hub genes in control (*n* = 10) *vs.* HF patients (*n* = 21) (* *P* < 0.05, ns: no significance).

The variation of mRNA level in hub genes ARNTL, PER3, CRY2, BHLHE41 and NPAS2 was detected using the peripheral blood of the participants by quantitative RT-PCR, which for CRY2 and BHLHE41 showed significant increase, whereas decrease for ARNTL and NPAS2 in the HF group in comparison to the control (*P* < 0.001); PER3 mRNA showed a slight higher expression in the HF group (*n* = 21) in comparison to the control group (*n* = 10), while no significant difference was found (*P* = 0.118) (Fig. 7), which is similar to the outcomes from our bioinformatics analysis.

## Discussion

Increasing evidence points to the association of circadian disruption with various diseases, not except for HF. Circadian rhythms-related genes (CCGs) generally show the differential expression in heart tissues, which may exert an essential role in HF initiation and progression, as demonstrated by mouse models (Lefta et al., 2012; Duong et al., 2019). Epidemiological evidence has also demonstrated the significant correlation of the disruption of circadian rhythms in humans with the increased risk of HF (Yamada et al., 2015; Li et al., 2021), that circadian rhythm disturbances may promote the development and progression of HF. As a result, there is an urgent requirement for a detailed understanding of the biological mechanism, serving as the reference for developing novel prevention, diagnosis, and treatment for HF.

In the present study, 10,163 DEGs were identified by a series of bioinformatics analyses based on gene expression profiles obtained from GSE57338, covering 4,615 up-regulated and 5,548 down-regulated genes in HF patients compared to healthy controls. After overlapping in the Circadian Gene DataBase (CGDB), we obtained 723 circadian rhythms-related DEGs. GO analysis indicated a function enrichment of circadian rhythms-related genes, such as regulating lipid metabolic processes, rhythmic processes, and circadian rhythm. Studies have validated the circadian rhythm-related lipid metabolism disorder as a critical step in HF development and progression (Wende et al., 2016; Crnko et al., 2019; Song et al., 2022), which reached an accordance to our bioinformatics analysis. Besides, the significantly enrichment of most circadian rhythms-related DEGs in AMPK signaling and the HIF-1 signaling pathway was revealed by the KEGG pathways analysis, in which AMPK serves as a primary regulator that integrates energy homeostasis with the circadian regulation of vital metabolic pathways (Jordan & Lamia, 2013; Lee & Kim, 2013).

Moreover, some studies have revealed the AMPK involvement in maintaining apoptosis, autophagy, and energy metabolism in degenerating hearts (Kops et al., 2002; Puigserver et al., 2003; Lagouge et al., 2006; Ronnebaum & Patterson, 2010; Schilling & Kelly, 2011; Kim et al., 2012). Hypoxia-inducible factor 1 (HIF1), an oxygen homeostasis regulator, has been validated to play a role in glucose metabolism and mitochondrial respiration (Peek et al., 2017), which could also be activated by the tissue hypoxia, showing the potential as an intriguing factor to mediate the relationship between metabolic energy disorder and HF (Fillmore, Mori & Lopaschuk, 2014; Warbrick & Rabkin, 2019). As outlined above, a metabolic shift is finely tuned by the metabolic energy sensors, including AMPK and HIF-1, which manipulate the cardiomyocyte molecular clock through the availability of energy and nutrient. The underlying mechanism of dysregulation of circadian rhythm could be associated with HF development and progression.

We selected eight overlapping genes as the most significant hub genes in GSE57338 according to the PPI network, which were, CRY1, ARNTL (also known as BMAL1), PER2, BHLHE41 (also known as DEC2), CRY2, PER3, NPAS2, and PER1. Further, ROC curves were plotted for evaluating the diagnostic values of hub genes, which identified five hub genes with high accuracy that showed AUC >0.7, that were, down-regulated (ARNTL, NPAS2) and up-regulated (PER3, CRY2, BHLHE41) genes in HF samples. Available data suggest that CLOCK (or NPAS2)-BMAL1/ARNTL heterodimer could activate the transcription of the negative elements, Period (Per1-3) and Cryptochrome (Cry1-2), as well as other clock-controlled genes that enable maximum advantage provided to cardiac function (Zhang & Jain, 2021). Our study demonstrates the association of HF with energy metabolism that is linked with circadian rhythm disorder. According to the study of Young et al., Cardiomyocyte-specific Bmal1 knockouts (CBK) mice exhibited metabolic dysfunction (depressed glucose utilization and increased fatty acid oxidation) (Young et al., 2014). Kohsaka et al. (2014) found that BMAL1 (-/-) mice tended to exhibit the mitochondrial dysfunctions in the heart. Moreover, the development of age-related dilated cardiomyopathy was also found in BMAL1 (-/-) mice, characterized by the thinned myocardial wall, dilated left ventricle, and the decreased cardiac output (Lefta et al., 2012). Neuronal PAS Domain Protein 2 (NPAS2) serves as an essential member of the clock gene that manipulates the biological clock’s rhythm. Studies have demonstrated a close relation of NPAS2 to cardiovascular diseases. For instance, Englund et al. (2009) found a close association with hypertension, which is also a risk factor for HF. A previous study has displayed a uniform converge of the primary dysregulated pathway(s) in the NPAS2 (-/-) animals on lipid metabolism (O’Neil et al., 2013), which is essential for the homeostasis maintenance of cardiac energy metabolism. In addition, NPAS2 (-/-) mice showed a lacked or blunted bimodal activity and sleep patterns in comparison to wild-type mice (Dudley et al., 2003). In contrast, Clock mutants of wild-type mice display the increased physical activity and food intake at rest and fasting (Turek et al., 2005), suggesting the nonredundant functions of Clock and Npas2 paralogs concerning energy balance (Huang et al., 2011).

Cryptochrome2 (CRY2) shows a 10-fold greater expression compared to CRY1 in the heart, which means that CRY2 is more involved in the heart’s clock negative feedback loop mechanism than CRY1 (Young, Razeghi & Taegtmeyer, 2001). Machicao et al. (2016) identified that CRY2 as a novel switch in hepatic fuel metabolism, which promotes the increased triglyceride storage and reduced glucose production, as well as to regulate muscle metabolism according to several studies (Ehrenborg & Krook, 2009; Jordan et al., 2017). Moreover, the genetic ablation of CRY1/2 exhibited an association to the exercise capacity increase in mice, implying it function to modulate metabolic flexibility in muscles, to achieve a balanced daily energy between supply and demand (Jordan et al., 2017). The clock repressor PER2 manipulates the lipid metabolism by modulating PPAR-gamma and mitochondrial rate-limiting enzymes. (Grimaldi et al., 2010; Neufeld-Cohen et al., 2016). It was demonstrated that the peak point of PER3 expression were positively correlated with physical activity and peak oxygen uptake in older adults (Takahashi et al., 2017). The stabilization of PER2 triggers a HIF-controlled metabolic switch that promotes the myocardial adaptation to ischemia (Eckle et al., 2012), confirming to the hypothesis that PER possibly optimizes the mitochondrial reaction to transformations in heart energy supply and request. BHLHE41, also known as DEC2, serves as a negative regulator of the circadian rhythm that suppresses Clock/Bmal1 expression (Honma et al., 2002). As reported, the dominant energy sensor phosphorylation of AMP-activated protein kinase (pAMPK) is markedly promoted in DEC knockout mice (Sato et al., 2016). The hypoxia-inducible factor-1*α* (HIF-1*α*) activates the transcription of Dec1 and Dec2 that play a role in regulating the lipid metabolism by inhibiting PPAR transcription (Yun et al., 2002; Sato et al., 2008; Park & Park, 2012; Chen & Yang, 2014). Consequently, the disruption of normal CCRGs expression may disturb the cardiac energy homeostasis, which might be related to the occurrence, development, and prognosis of HF (Yan et al., 2021). Based on the integrated bioinformatical studies, we conducted a comprehensive and novel analysis of clock gene expression profiles in patients with HF. In particular, the ARNTL, CRY2, BHLHE41, and NPAS2 are identified as the potential biomarkers associated with the circadian rhythm in HF. Meanwhile, the down-regulated genes, ARNTL and NPAS2, and the up-regulated genes, CRY2 and BHLHE41, could serve as the promising therapeutic targets for HF. Additionally, the significantly altered differential genes involved in AMPK and HIF-1*α* pathways were found in HF patients. The detection of clock genes involved in HF as a highly relevant topic that will exert implications for research and translational value with its diagnostic relevance.

There exist several limitations in the present study. We employed the data from publicly available databases, which resulted in a proneness to selection bias of the analysis. The DEGs were identified in the GSE57338 dataset, which provided the study samples mainly from heart tissue, while we employed it to validate the hub genes obtained from peripheral blood. It is required to verify the expression of hub genes in heart tissue samples, which may lead to the more reliable results. In addition, the number of enrolled patients was relatively limited here, which may affect study results to a certain extent. Therefore, more extensive prospective studies are required, as well as further experimental investigation to more comprehensively understand how ARNTL, CRY2, BHLHE41, and NPAS2 contribute to HF pathogenesis.

## Conclusions

Our study demonstrated ARNTL, CRY2, BHLHE41 and NPAS2 as potential biomarkers of circadian rhythm in HF, with the potential to provide novel markers or targets for the diagnosis and treatment of HF.

##  Supplemental Information

10.7717/peerj.14734/supp-1 Supplemental Information 1 Primer sequences

10.7717/peerj.14734/supp-2 Supplemental Information 2 Raw data of RT-qPCR

## Additional Information and Declarations

### Data Deposition

The following information was supplied regarding data availability: The data is available at NCBI GEO, accession numbers: GSE57338, GSE5406. The raw measurements are available in the Supplementary Files.
