# Supplementary material for: Identification of the potential biomarkers associated with circadian rhythms in heart failure
Source: PeerJ. 2023 Jan 20;11:e14734. doi: 10.7717/peerj.14734 (PMC9869779; doi:10.7717/peerj.14734)
Supplement: Supplemental Information 1 [file peerj-11-14734-s001.docx]

**Table S1. Primer sequences**

| Gene | Primer Sequence | |
| --- | --- | --- |
| ARNT1 | 5′-TGCAACGCAATGTCCAGGAA-3′ (forward) | 5′-GGTGGCACCTCTTAATGTTTTCA-3′ (reverse) |
| BHLHE41 | 5′-GGTCTCCCAAGCCTACCGTC-3′ (forward) | 5′-GGTATCCTTGGTGTCGTCTCGT-3′ (reverse) |
| CRY2 | 5′-GGAGAACCACGACGAGACCTAC-3′ (forward) | 5′-ACAACCAAAGCGCAGGTAGG-3′ (reverse) |
| NPAS2 | 5′- GCAGTCTGTCTGAGTCGTCAGG-3′ (forward) | 5′-GACCGACGATGCCTACACG-3′ (reverse) |
| PER3 | 5′- AAGAGGTCGCTCCAGCCC-3′ (forward) | 5′- GCCATAGAAAGCGGTGACTGA-3′ (reverse) |
